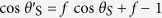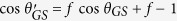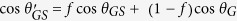# Solving the Controversy on the Wetting Transparency of Graphene

**DOI:** 10.1038/srep15526

**Published:** 2015-10-26

**Authors:** Donggyu Kim, Nicola M. Pugno, Markus J. Buehler, Seunghwa Ryu

**Affiliations:** 1 Department of Mechanical Engineering, Korea Advanced Institute of Science and Technology (KAIST), Daejeon, Republic of Korea; 2 Laboratory of Bio-Inspired and Graphene Nanomechanics, Department of Civil, Environmental, and Mechanical Engineering, University of Trento, Trento, Italy; 3 Centre for Materials and Microsystems, Fondazione Bruno Kessler, Via Sommarive 18, I-38123 Povo (Trento), Italy; 4 School of Engineering and Materials Science, Queen Mary University of London, Mile End Road, E1 4NS, London, UK; 5 Department of Civil and Environmental Engineering, Massachusetts Institute of Technology, Cambridge, USA

## Abstract

Since its discovery, the wetting transparency of graphene, the transmission of the substrate wetting property over graphene coating, has gained significant attention due to its versatility for potential applications. Yet, there have been debates on the interpretation and validity of the wetting transparency. Here, we present a theory taking two previously disregarded factors into account and elucidate the origin of the partial wetting transparency. We show that the liquid bulk modulus is crucial to accurately calculate the van der Waals interactions between the liquid and the surface, and that various wetting states on rough surfaces must be considered to understand a wide range of contact angle measurements that cannot be fitted with a theory considering the flat surface. In addition, we reveal that the wetting characteristic of the substrate almost vanishes when covered by any coating as thick as graphene double layers. Our findings reveal a more complete picture of the wetting transparency of graphene as well as other atomically thin coatings, and can be applied to study various surface engineering problems requiring wettability-tuning.

Graphene has been a subject of intense research on the basis of its superior optical transparency, electrical conductivity, and mechanical strength^1^,^2^,^3^,^4^,^5^,^6^. Recently, Rafiee *et al.* reported another superior property of graphene, “wetting transparency”, which implies that the van der Waals (vdW) interaction between graphene and any liquid placed on top of it is negligible, allowing the “transmission” of the substrate contact angle above graphene. The graphene wetting transparency was spotlighted because of its versatile potential applications^7^,^8^,^9^,^10^. Yet, Shih *et al.*^11^ showed that the contact angle is significantly affected by the vdW interaction of monolayer graphene and the wetting of the substrate is partially transmitted only for substrates with moderate contact angles (40° ~ 90°). With addition of more graphene layers, the wetting transparency diminishes as the vdW interaction by graphene becomes dominant over the vdW interaction by the substrate.

Although Shih *et al.*^11^ established a framework to understand and analyze the wetting transparency, the theory can be improved by incorporating two critical factors that are very important in modeling real experiments. First, the Boltzmann distribution was used to model the water density profile without considering the bulk modulus, a measure of resistance to hydrostatic compression, of the liquid. If a few mathematical flaws are corrected, the theory predicts unrealistically high water density (up to an order of magnitude larger than the ambient density) above the graphene-covered hydrophilic surface. Second, while the theory considered a completely flat surface, it can be improved to explain a wide range of experimental data for surfaces with unknown roughness, if various wetting modes are concerned.

In this work, we report a unifying framework to account for the bulk modulus of the liquid as well as the surface roughness to realistically describe the wetting phenomena. First, we correct a few mathematical mistakes of the previous work in calculating the van der Waals (vdW) interaction energy between the liquid and the substrate. With the corrections, the previous theory predicts zero contact angle for any hydrophilic solid covered by graphene, i.e. complete breakdown of wetting transparency. By adjusting the contact distance between substrate and liquid, the theory can fit experimental data supporting partial wetting transparency (See Supplementary Information). Still, the theory suffers from the prediction of unrealistically high liquid density because the intermolecular interactions among liquid molecules were disregarded. We employ the hydrostatic equation coupled with the experimental water density-pressure curve to correctly capture the intermolecular interaction by incorporating the bulk modulus in computing the liquid density profile. With aforementioned corrections, we show that our theory explains the experimental results for flat substrates. In addition, we extend the theory for substrates with roughness^7^,^11^,^12^,^13^ using various conventional wetting modes^14^ as well as for substrates covered with multi-layered graphene sheets.

## Results

### Accurate evaluation of the van der Waals interaction energy for flat surfaces

**Figure 1:**
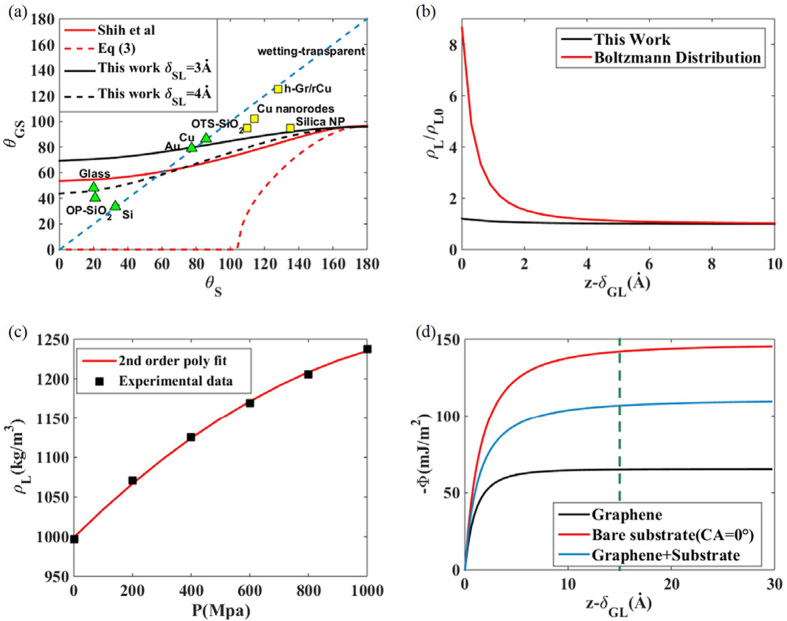
Theoretical predictions about wetting phenomenon of graphene on the flat substrate. (**a**) Predicted **θ**_**GS**_ as a function of **θ**_**S**_ depicting the previous results^11^ (red), the results with vdW math correction (red dotted), and the results with both vdW and bulk modulus corrections (black). Triangles and squares represent experimental data from the literature^7^,^11^,^12^,^13^ (**b**) Liquid densities near the bottom of the liquid droplet predicted without (red) and with (black) bulk modulus consideration (**c**) Density of a compressed liquid as a function of pressure^17^ (**d**) vdW potential energy per unit area stored in the volume from the bottom of the liquid droplet to the height of **z − δ**_**GL**_.

The contact angle θ can be determined by the Young-Dupre equation^15^,^16^, 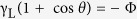, where γ_L_ refers to the liquid surface tension. The vdW interactions per unit area between liquid and substrate, Φ, can be computed from 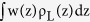 where w(z) refers to the vdW interaction between one liquid molecule and the substrate, 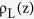 is the liquid density profile, and z is the distance between the molecule and the substrate. Monolayer graphene is transparent if Φ_S_ for any bare solid is identical to Φ_GS_ for the graphene-covered surface. Figure 1a shows the relationship between the contact angle on the monolayer graphene-covered surface, **θ**_**GS**_, and the contact angle on the bare solid, **θ**_**S**_. The previous study^11^ revealed that the lower bound of Φ_GS_ is the Φ for free standing monolayer graphene, which implies that the upper bound of **θ**_**GS**_ is the contact angle of the monolayer graphene (Fig. 1a). However, a higher contact angle (**θ**_**GS**_ > 120°) was reported for the conformal graphene film on a rough copper surface^12^. Also, if the mathematical errors in vdW interaction is corrected (see Methods), the theory predicts **θ**_**GS**_ = 0° for any solid with **θ**_**S**_<105° (Fig. 1a), indicating a complete breakdown of wetting transparency. It does not mean that the main conclusion of the previous study is incorrect, because the theory can still fit to experimental data with the modification (See Supplementary Information).

To understand the origin of the discrepancy, we plot the liquid density profile as a function of the distance z from the graphene layer in Fig. 1b. When the bare solid having a contact angle of 45° is covered with a monolayer graphene, the liquid density at z = δ_GL_ is predicted to be about 8 times the density *ρ*_*L*0_ at the ambient conditions. Here, δ_GL_ refers to the equilibrium distance between the bottom of liquid and the graphene layer. The peak density of 8*ρ*_*L*0_ is unphysical because water solidifies at a much lower density^17^. Also, the highest water density was reported to be 1.5 ~ 2.5*ρ*_*L*0_ in molecular dynamics simulations^18^,^19^,^20^. The density profile was significantly overestimated because it was computed by 
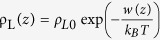
 without considering the intermolecular interaction among liquid molecules. Instead of the intermolecular potential that cannot be computed analytically, the bulk modulus can be incorporated in computing the density profile to account for the resistance to hydrostatic compression.

In order to correctly account for the liquid bulk modulus, we compute the density profile by coupling a hydrostatic equation, 
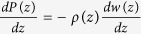
 where P(z) is the pressure profile of liquid, with the experimental pressure-density relationship of the water from 1 atm to 1 GPa (the maximum pressure of liquid on the super hydrophilic substrate) measured at room temperature (Fig. 1c)^21^. Beyond 1GPa, water solidifies^17^. The pressure-density relationship can be fitted to the 2^nd^ order polynomial 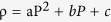. The bulk modulus 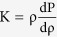 cannot be considered as a constant for the wide range of pressure considered in this study. Combining the hydrostatic equation and the pressure-density relationship, we can obtain the liquid profile P(z) as a function of z from


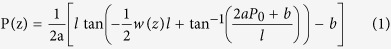


where 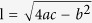 and P_0_ = 1 atm. After taking the bulk modulus into account, we obtain a realistic prediction of density as depicted in Fig. 1b. In addition, we set the solid-graphene equilibrium distance *δ*_*GS*_ as 3 Å and the solid-water distance *δ*_*SL*_ as 3 ~ 4 Å to match the reported values^5^,^6^,^22^, both of which were set to excessively large in the previous work (see Supplementary Information). We note that the density profile can be further improved by incorporating the finite size of the liquid droplet and density oscillation in the z direction observed in molecular dynamics^18^,^19^,^20^. With the density correction, the theoretical prediction agrees well with the experimental contact angle measurements with flat substrates (Fig. 1a). Most contact angle measurement data for flat substrates^7^,^11^ lie between the two curves with δ_SL_ = 3 Å and δ_SL_ = 4 Å, which implies that monolayer graphene has a limited wetting transparency for hydrophilic surfaces.

### Prediction of contact angles for rough surfaces

**Figure 2:**
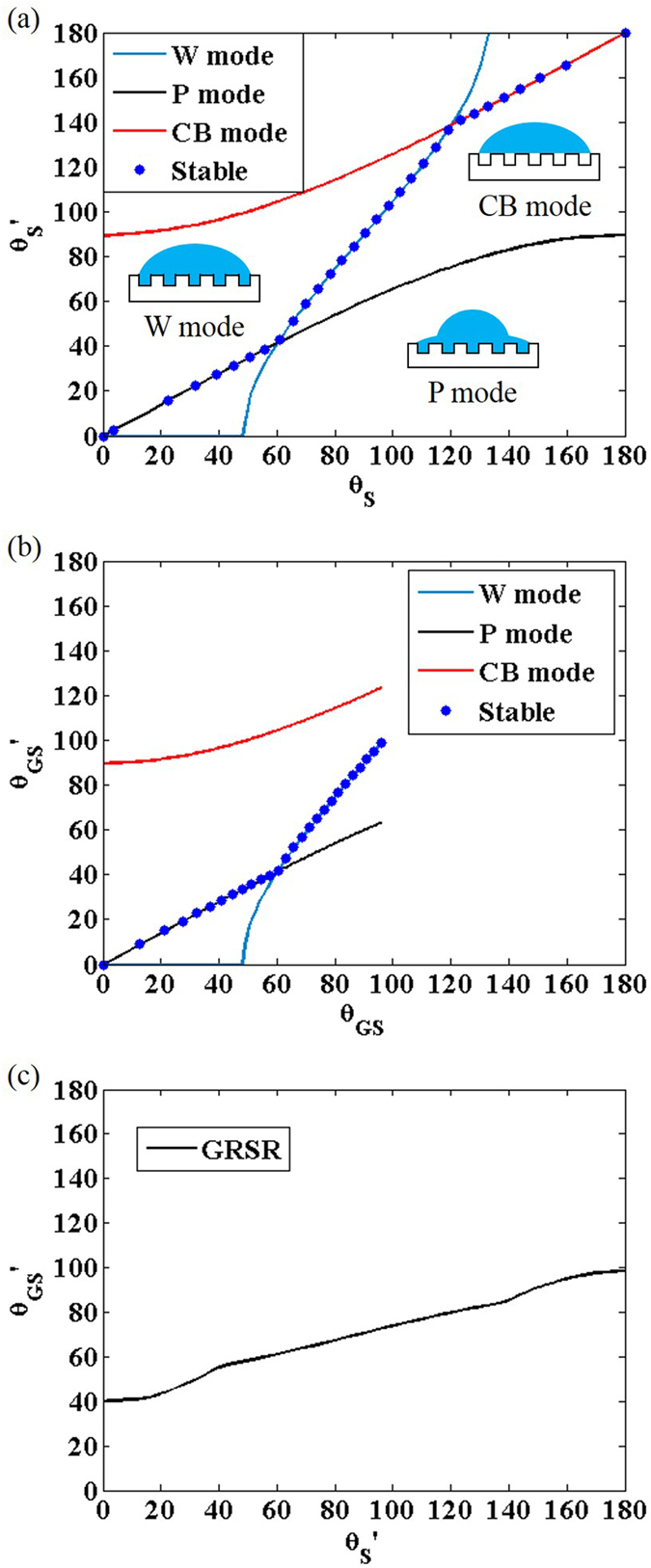
Illustration of the contact angle calculation for graphene-covered rough surface for the GRGR model with r = 1.5 and *f* = 0.5. (**a**) Conventional possible wetting modes of the bare substrate, and the actual path of which possesses the minimum free energy^14^ (**b**) Conventional possible wetting modes of the graphene covered substrate and the actual path. Note that ***θ*****′**_***GS***_ cannot exceed the contact angle of graphene (96°) (**c**) Contact angle change due to the existence of a graphene film of GRSR model.

A gap nonetheless remains between the experimental measurements and the theoretical predictions for hydrophobic surfaces. Since experiments with **θ**_**S**_ ≥ 90° were conducted on rough substrates, we analyze the wetting on bare rough surfaces by the conventional wetting theory with three possible wetting modes: penetration (P), Wenzel (W), and Cassie-Baxter (CB) states (Fig. 2a)^14^. We then model the graphene-covered rough substrate either by a rough graphene sheet on a rough substrate (GRSR) model or a flat graphene sheet on a rough substrate (GFSR) model. The former considers conformal contact made between a graphene layer and moderately rough surfaces, whereas the latter describes non-conformal contact on severely rough surfaces. From the conventional wetting theory on a rough surface, we obtained the contact angles ***θ*****′**_***GS***_ of the GRSR and GFSR models as functions of the inherent graphene film contact angle **θ**_**G**_, the contact angle of a graphene covered flat substrate **θ**_**GS**_, roughness r, and the fraction of bare substrate area wet by the liquid *f*, as presented in Table 1. For GRSR model, the ***θ*****′**_***GS***_ formulae are derived by treating the graphene-covered substrate as a single material with contact angle **θ**_**GS**_, because GRSR model considers conformal contact between graphene film and substrate. For GFSR model where only CB mode is available for the flat surface, the formula is derived by the CB mode for composite surface. A fraction *f* of liquid molecules interacts with both graphene and the substrate, while the other fraction 1 − *f* interacts only with graphene.

The conventional wetting theory holds only if the surface texture has a certain characteristic length scale in roughness because it assumes that the liquid-solid interfacial energy is given by the product of the bulk liquid-solid interfacial energy and the effective area of liquid-solid contact^14^. For example, the conventional Wenzel theory is not applicable to atomically rough surfaces composed of steps and adatoms only. We find that the vdW potential energy per area converges more than 90% within 15 Å from the bottom of the liquid for an extremely superhydrophilic substrate having a zero contact angle, as depicted in Fig. 1d. The vdW potential will converge within shorter distance for substrates with higher contact angles. Therefore, the conventional wetting theory would hold if the characteristic scale of roughness is greater than a few nanometers. Noting that the characteristic length of the rough surfaces is at least a few tens of nanometers in experiments, the conventional wetting theory can be applied to analyze the experiments.

We obtain the relation between **θ**_**GS**_ and **θ**_**S**_ for the flat surface by calculating the Φ_GS_ and Φ_S_ (see **Methods** for details). As in the previous study^11^, we tune **θ**_**S**_ from 0° to 180° by gradually increasing the value of ρ_S_*A*_*SL*_ from 0 to 11.7 *eV*Å^3^, and compute ***θ*******_***GS***_ for the corresponding range of ρ_S_*A*_*SL*_ to get **θ**_**GS**_** − θ**_**S**_ relationship. Then, the equilibrium wetting mode on the rough surface is determined by the minimum free energy principle, as demonstrated for the GRSR model with r = 1.5 and f = 0.5 in Fig. 2. On the hydrophilic surface (θ < 90°), the equilibrium angle is given by the higher contact angle between the predictions by P mode and W mode. In contrast, the equilibrium angle on the hydrophobic surface (θ > 90°) is chosen as the lower angle between the predictions by W mode and CB mode^14^. According to this principle, we calculate the equilibrium angles ***θ*****′**_***S***_ on the rough bare substrate (Fig. 2a) and **θ′**_**GS**_ on the rough graphene-covered substrate (Fig. 2b). Combining the two predictions, we can plot the relationship between ***θ*****′**_***S***_ and ***θ*****′**_***GS***_ (i.e. pairs of ***θ*****′**_***S***_** − *****θ*****′**_***GS***_ that correspond to identical ρ_S_*A*_*SL*_ values), as depicted in Fig. 2c.

**Figure 3:**
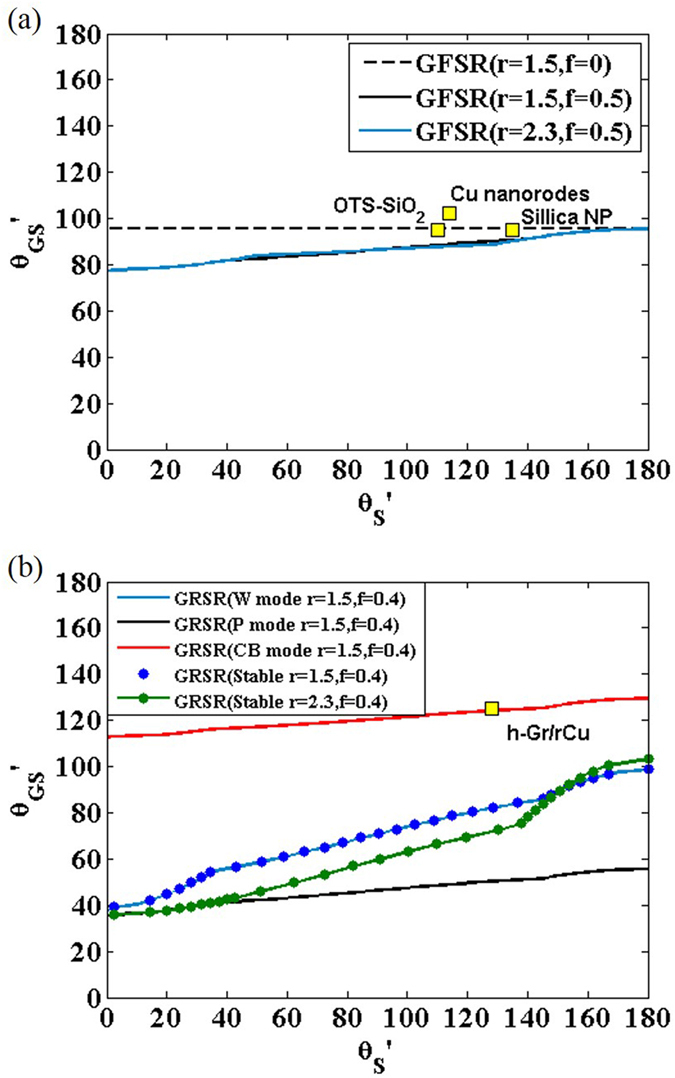
Theoretical predictions of contact angles of graphene on a rough substrate. (**a**) Predicted ***θ*****′**_***GS***_ as a function of ***θ*****′**_***S***_ for GFSR model^11^,^13^. The measurements lie within the curves with *f* ≤ 0.5 regardless of the choice of r. (**b**) Predicted ***θ*****′**_***GS***_ for possible wetting modes as a function of ***θ*****′**_***S***_ for GRSR model^12^. The data cannot be explained by the equilibrium curve because the upper bound of ***θ*****′**_***GS***_ is **103.5°** within the reasonable r range of **1 ≤ r ≤ 2.3**^23^.

To validate the theory for a rough substrate, we compare the theoretical predictions with experimental results^7^,^11^,^12^,^13^. Since the experiments were conducted on surfaces with unknown roughness, we freely choose r within the range of a few available direct measurements by white light profilometry and scanning electron microscope^23^ (1 for flat surface ≤ r ≤ 2.3 for very rough surface), and f within the definition (fraction of bare substrate area wet by the liquid, i.e. 0 < *f* ≤ 1). It is known that a graphene layer floats on the surface when graphene grown by another surface is transferred^13^. Therefore, we applied the GFSR model for most of the data points including Silica NP, Cu nanorodes, and OTS-SiO2, as depicted in Fig. 3a. We obtain the relation between ***θ*****′**_***S***_ and ***θ*****′**_***GS***_ by following the procedure used in Fig. 2 except that we only considered CB mode when computing ***θ*****′**_***GS***_. Most rough substrates are found to have a contact angle around the contact angle of monolayer graphene 96°, the upper bound for the GFSR mode. The measurements lie within the curves with *f* ≤ 0.5 regardless of the choice of r. The contact angle above 96° can be explained only if we consider a more complex model in which rough graphene is non-conformally adhered to the rough substrate (see Supplementary Information).

On the other hand, if a graphene film is directly grown on a rough substrate, the film does not float on the substrate but rather conformally adheres to the rough substrate^12^. We compare the measured contact angle of the conformal graphene film on a rough copper surface (denoted as h-Gr/rCu) with the GRSR model and obtain a very interesting result. While the equilibrium contact mode is predicted to be the W-mode, the predicted equilibrium contact angle curve, ***θ*****′**_***S***_** − ***θ*****′**_***GS***_** (the blue or green line in Fig. 3b), shows a large discrepancy with the measurement. The data cannot be explained by the equilibrium curve because the upper bound of equilibrium ***θ*****′**_***GS***_ is 103.5° within the reasonable r range of 1 ≤ r ≤ 2.3^23^. Interestingly, the experimental result lies on the angle from CB mode with *f* = 0.4. We suspect that the droplet forms the metastable CB mode because of the energy barrier for the transition from the CB state to the air-free W state^14^. Indeed, the original experimental paper^12^ also speculated that the high contact angle originated by the CB mode.

### Wetting transparency of multilayer graphene

**Figure 4:**
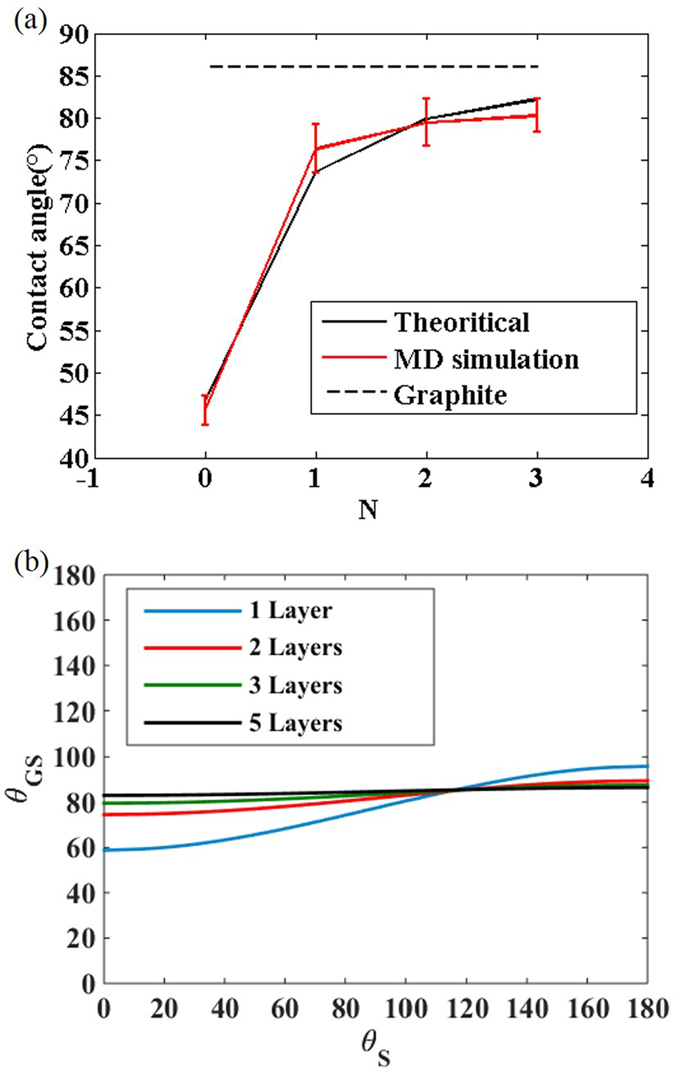
Theoretical and MD simulation result for wetting of multilayer graphene on a substrate. (**a**) Predicted **θ**_**GS**_ for composition of N layers of graphene film and solid substrate (**θ**_**S**_ = 45°) (**b**) Predicted **θ**_**GS**_ as a function of **θ**_**S**_ for composition of 1 ~ 5 layers of graphene film and solid substrate.

We have also studied the wetting transparency of multilayer graphene and find the implication on the wetting transparency of atomically thin coatings. The contact angle of the N-layered graphene covered substrate **θ**_**NGS**_ dramatically converges to the contact angle of graphite 86°^11^ as the number of graphene films increases. We reveal that **θ**_**NGS**_ is not substantially affected by **θ**_**S**_ if more than two layers of graphene films are stacked (Fig. 4a,b). This phenomenon can be analyzed by comparing the distance between the substrate and the water molecules. When monolayer graphene is covered on a substrate, the distance between the substrate and the bottom of the liquid is 6.28 Å, and the substrate contributes a non-negligible portion of the vdW interaction between the entire surface and the liquid. The distance becomes more than 9 Å if two layers of graphene are stacked. Except for with super-hydrophilic solid substrates, double graphene layers will contribute the dominant portion of the vdW potential. If even more graphene layers are stacked, the influence of the solid substrate almost vanishes and the contact angle converges to the contact angle of graphite. Our theory is validated by molecular dynamics simulations^24^ (see Methods for details). We consider a model substrate having a contact angle of 45°, and measure the contact angle change when 1–3 graphene layers are covered. The molecular dynamics simulations confirm that the contact angle converges very quickly when three or more layers of graphene are stacked (Fig. 4a).

Our finding implies that most related 2D materials would not show any wetting transparency effect. Due to corrugated or puckered structures, monolayer silicene or phosphorene sheets are significantly thicker than the monolayer graphene^25^. Typical transition metal dichalcogenides materials such as MoS_2_ is 6.5 Å thick^26^, which is comparable to the thickness of graphene double layer. Thus, related 2D materials would show very limited wetting transparency expected for double layer graphene. Any atomically thin coating with the thickness comparable to graphene double layer is enough to almost erase the wetting characteristic of the bare solid.

## Discussion

Based on the theoretical and computational analyses, we provide a comprehensive view on the wetting transparency of graphene. At a first glance, the contact angle measurements on bare and graphene-covered surfaces can be interpreted as an evidence for the wetting transparency for a wide range of contact angles (20° ~ 120°) because they scatter around the perfect wetting transparency line. We show that the observed correlation is a coincidence and cannot be explained by a single universal curve. When a flat graphene layer is covered on a flat surface, the wetting behavior can easily be understood by computing the vdW interaction with the liquid modulus effect. The upper bound of **θ**_**GS**_ is given by the contact angle of the free standing graphene (96°), and we can conclude that flat graphene monolayer is partially transparent for hydrophilic surfaces. However, in the presence of the surface roughness, three different wetting modes must be considered to determine the equilibrium as well as metastable contact mode to explain the experimental data. When a flat graphene layer is covered on a rough surface, the contact angle of the free standing graphene becomes the upper bound of **θ**_**GS**_. For a wrinkled graphene (with characteristic roughness scale larger than 10 nm) placed on rough bare surfaces, a contact mode with higher **θ**_**GS**_ can be formed. In addition, we show that the wetting transparency of graphene double or more layer is negligible, which implies that multilayer graphene covered surface can be treated as a pure graphite surface in terms of its wetting characteristics.

In conclusion, we study the wetting transparency of graphene by accurately calculating the vdW interaction energy for various wetting modes. We reveal that the observed partial wetting transparency cannot be explained without accounting for the bulk modulus and the surface roughness. We also find that this partial wetting transparency almost vanishes for double or more layers of graphene sheets. A natural extension of our work is the research on ionized substrates where coulomb interaction becomes significant, and more complex rough surfaces. We believe that our study can provide a more complete picture of the seemingly partial wetting transparency of monolayer graphene and can be applied to understand wetting phenomena in various circumstances.

## Methods

### Correction of mathematical flaws in the previous work

First, we briefly review the vdW ineraction calculation presented in Shih *et al.*^11^ with the same notations, and then correct a few mathematical flaws. The attractive vdW interaction potential between one carbon atom and one liquid molecule is given by 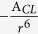, where r and A_*CL*_ are the distance and the vdW parameter between the two entities. Summing up the vdW potential from all pairs of a liquid molecule and carbon atoms, the vdW interaction between one liquid molecule and a flat, infinitely large monolayer graphene sheet is given by 
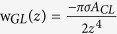
, where 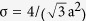 is the surface density of carbon atoms (with the graphene lattice constant a = 2.49 Å) and z is the shortest distance between the liquid molecule and the graphene plane. The vdW interaction for a flat, infinitely large N-layer graphene sheet adds up to 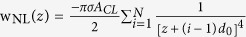, where d_0_=3.35 Å is the interlayer distance between the graphitic planes. Ignoring the bulk modulus of the liquid, the density profile of liquids molecule were computed from the Boltzmann distribution, 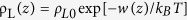.

The first mistake in the previous work was made in the calculation of the total vdW interaction potential per unit area between the liquid and the contacting N-layer graphene by 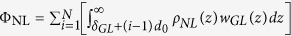. The density profile ρ_NL_(*z*) was written as 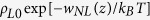, and 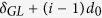 referred to the shortest distance between the i^th^ layer and the liquid. The shortest distance *δ*_*GL*_ between the uppermost graphene and the liquid was set to be 3.28 Å, according to the MD results. However, the integral in the square brackets underestimated the vdW interaction from all layers except i = 1, because the liquid density at the bottom (i.e. at 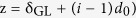, was underestimated as 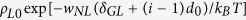, although it must be 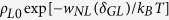, regardless of i. Thus, Φ_NL_ must be corrected as


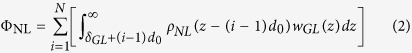


where 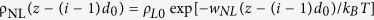.

For a similar reason (see Supplementary Information), the total vdW interaction per unit area Φ_*SNL*_ between the liquid and a sheet of N-layer graphene supported by a solid substrate was incorrectly computed and must be fixed as follows:


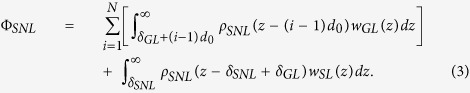


Here, 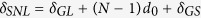 is the shortest distance between the liquid and the solid substrate, where *δ*_*GS*_ is the equilibrium contact separation between graphene and the solid. *ρ*_*SNL*_(*z*) refers to the liquid density profile above the entire surface, and 
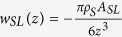
 to the vdW interaction between a liquid molecule and the solid substrate. ρ_S_ is the density of the substrate and A_SL_ is the vdW interaction parameter between one solid atom and one liquid molecule. On the other hand, the vdW interaction per unit area between the liquid and the bare solid was calculated without any mathematical error by 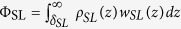, where the liquid density profile is defined by 
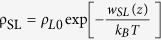
 and *δ*_*SL*_ is the distance between the liquid and the bare solid. **θ**_**S**_ is tuned from 0° to 180° by gradually increasing the value of ρ_S_*A*_*SL*_ from 0 to 11.7 *eV*Å^3^, and **θ**_**GS**_ is also computed for the corresponding range of ρ_S_*A*_*SL*_.

### Molecular dynamics simulations

We stack graphene films on an arbitrary solid substrate with the contact angle 45°, and calculate contact angles as we add more graphene layers on top (Fig. S1a). We prepare 16,000 liquid water molecules and use half-cylindrical shaped liquid droplet to remove the size effect from triple junction^7^. The simulations are carried out in 300K NVT ensemble using LAMMPS package with a time step of 1 fs. The liquid water molecules are modeled by the extended simple point charge model and the bond lengths are constrained by SETTLE algorithm^11^. On the other hand, substrate is modeled by uncharged vdW particles in face-centered-cubic crystal. For simplicity, atoms in graphene layers and substrate are fixed during simulation. We use 1.5 nm cutoff distance when computing the vdW interactions, and pppm method is used for calculating the coulomb interactions. We equilibrate the molecules for 1 ns and collect the contact angle from the boundary of the liquid droplet obtained from the local water density during the following 1 ns (Fig. S1b)^24^.

## Additional Information

**How to cite this article**: Kim, D. *et al.* Solving the Controversy on the Wetting Transparency of Graphene. *Sci. Rep.* **5**, 15526; doi: 10.1038/srep15526 (2015).

## Supplementary Material

Supplementary Information

## Figures and Tables

**Table 1:** Contact angles of three roughness models of graphene covered solids (Bare substrate, GRSR, GFSR) due to the wetting modes.

	Bare substrate	GRSR	GFSR
Penetrate mode	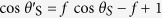	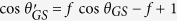	—
Wenzel mode	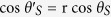	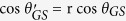	—
Cassie Baxter mode