# Supplementary material for: Solving the Controversy on the Wetting Transparency of Graphene
Source: Sci Rep. 2015 Oct 26;5:15526. doi: 10.1038/srep15526 (PMC4620452; doi:10.1038/srep15526)
Supplement: Supplementary Information [file srep15526-s1.pdf]

Supplementary Information

Solving the Controversy on the Wetting Transparency of Graphene

Donggyu Kim [1], Nicola Pugno [2,3,4], Markus J. Buehler[5], and Seunghwa Ryu [1,*]

Supplementary Figure1: Snapshot of MD Simulation

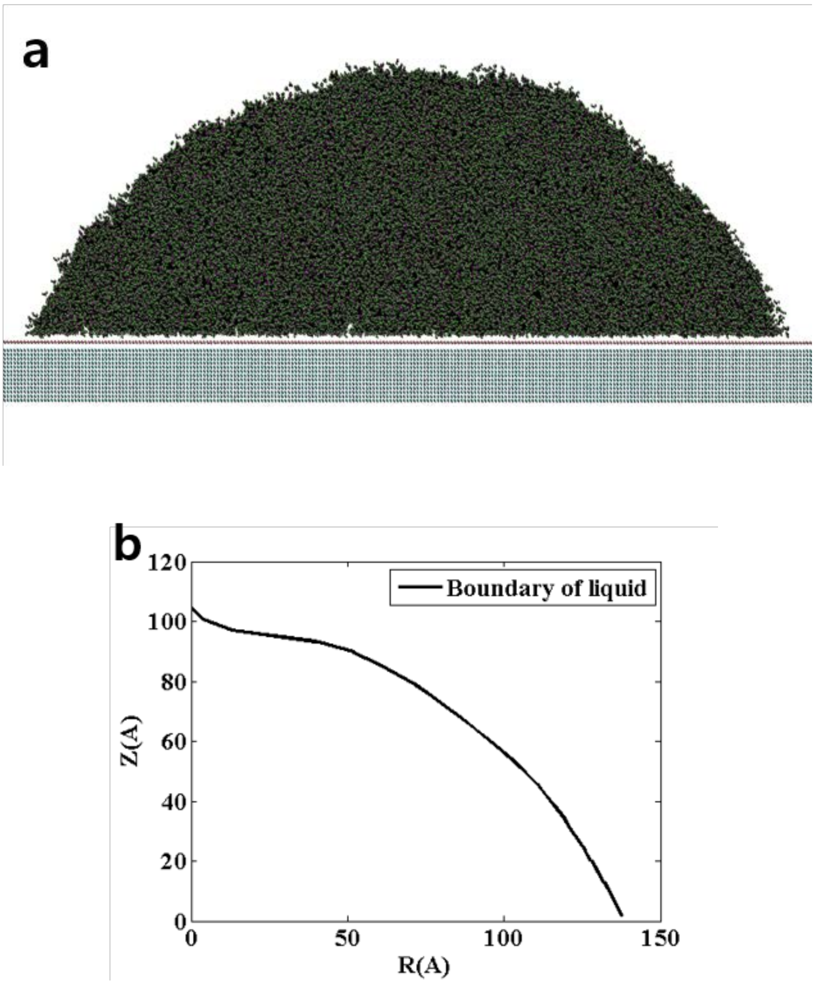

Supplementary Figure S1 (a) Snapshot image of MD simulation of wetting phenomenon on 1layer graphene + solid substrate (b) Average boundary contour of liquid droplet of 100 snapshots estimated by local density[1]

Supplementary Figure2: The Original and Corrected Results by Shih et al.[2] with Modified Values of δ_{SL}

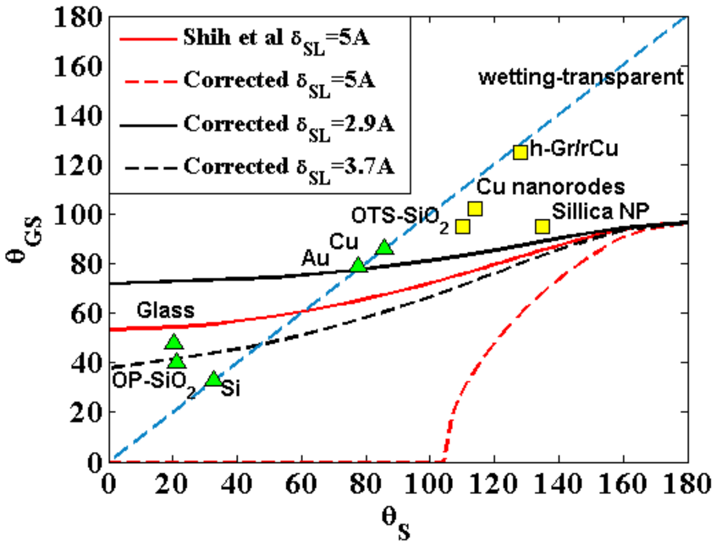

Supplementary Figure S2. The original result by Shih et al.[2] with δ_{SL}=5Å (red solid) and corrected results with various values of δ_{SL}. With the corrections of mathematical flaws, the prediction deviates far from experimental data set (red dotted). However, with the value of δ_{SL}=2.9~3.7Å, the theory suggested by Shih et al. can predict experimental data (black solid and black dotted). Thus, the qualitative conclusion that graphene is translucent to wetting remains valid. The significant density overestimation problem persists with the modification of δ_{SL} because the intermolecular interaction among liquid molecules is disregarded in the previous study.

Supplementary Figure3: GRSR2 Wetting Model of Graphene Covered Substrates

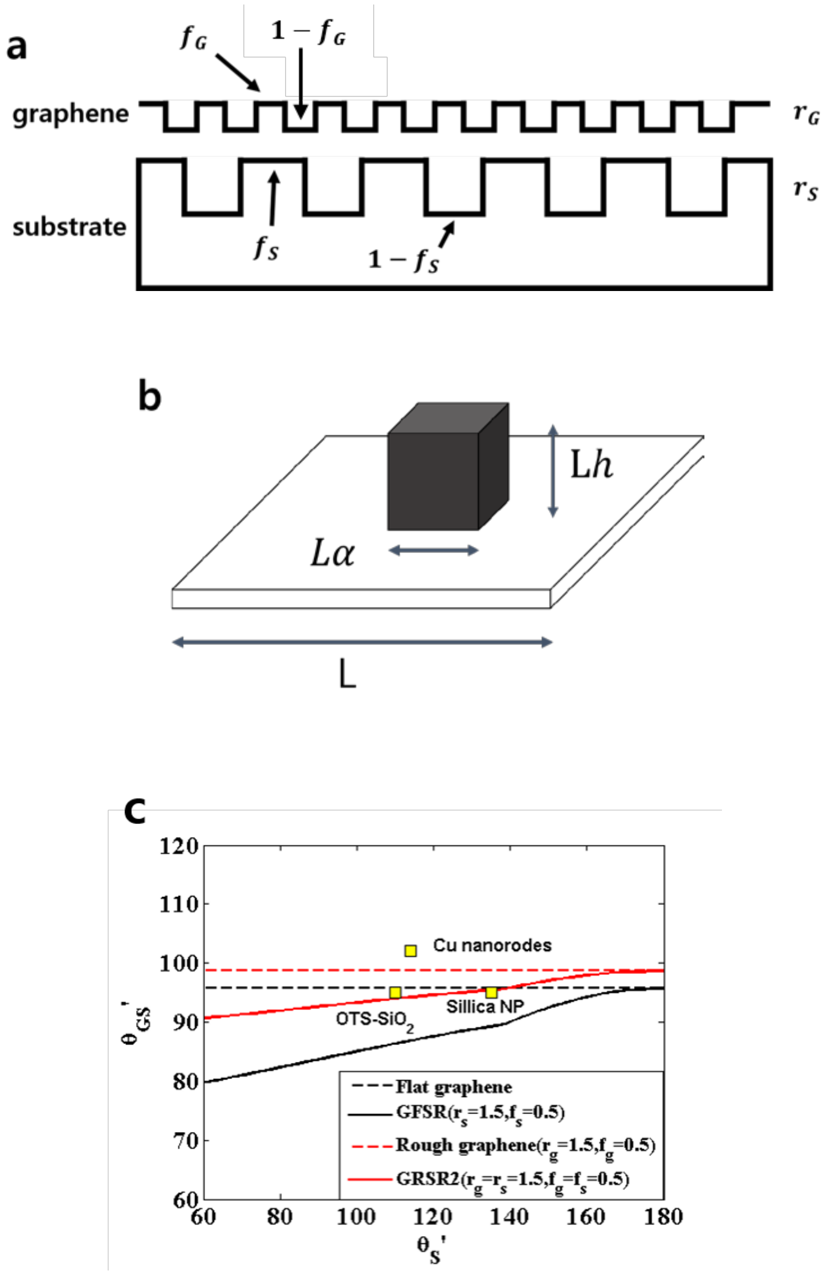

Supplementary Figure S3 (a) Configuration of the GRSR2 wetting mode which considers that rough graphene is non-conformally adhered to rough substrate (GRSR2). $\mathbf{r_g}$ and $\boldsymbol{f_g}$ refer to roughness of graphene, while $\mathbf{r_s}$ and $\boldsymbol{f_s}$ refer to roughness of substrate. (b) The texture of rough surfaces when we develop the contact angle formulae for GRSR2 model. We considered a periodic array of such post with vertical side walls. (c) The relation between $\boldsymbol{\theta'_{GS}}$ and $\boldsymbol{\theta'_S}$ predicted by GRSR2 model and GFSR model. GRSR2 model predicts the contact angle closer to the experimental data.

Supplementary Note 1: The vdW Interaction between Liquid and Multilayer Graphene Covered Substrate

A similar mistake was repeated when evaluating the total vdW interaction per unit area Φ_{SNL} between the liquid and a sheet of N-layer graphene supported by a solid substrate. The vdW interaction between one liquid molecule and the entire substrate, $\mathrm{w_{SL}}(z)$ was evaluated by integrating the vdW potential of one liquid and one solid molecule in half-infinite space as $\mathrm{w_{SL}}(z) = \rho_s \int_z^{\infty} (\int \frac{-A_{SL}}{r^6} d\Sigma) d\zeta$. Here ρ_s refers to density of the solid substrate, Σ to infinite surface which is perpendicular to z direction, ζ to coordinate variable along z direction and Shih et al.[2] adjusted the value of $\rho_S A_{SL}$ to represent various solid substrate. Thereafter, the water density profile above the entire surface $\rho_{SNL}(z)$ was computed by $\rho_{L0} \exp\left[-\frac{w_{SNL}(z)}{k_B T}\right]$ with $\mathrm{w}_{SNL}(z) = w_{NL}(z) + w_{SL}(z + \delta_{SNL})$ where z=0 referred to the top of N-layer graphene. Although Shih et al. wrote this equation as $\mathrm{w}_{SNL}(z) = w_{NL}(z) + w_{SL}(z)$, we find that δ_{SNL} is necessary to reproduce their data. $\delta_{SNL} = \delta_{GL} + (N-1)d_0 + \delta_{GS}$ is to the shortest distance between the liquid and the solid substrate, where $\mathrm{d_0}$ is the distance between graphene layers and δ_{GS} is the equilibrium contact separation between graphene and the solid. However, the term $w_{SL}(z + \delta_{SNL})$ must be replaced by $w_{SL}(z + \delta_{SNL} - \delta_{GL})$ to correctly account for the shortest distance between the N-layer graphene and the solid substrate. Also, the total vdW interaction potential per unit area between liquid and the entire surface, ϕ_{SNL}, was computed by

$$\Phi_{\mathrm{SNL}} = \sum_{i=1}^{N} [\int_{\delta_{GL}+(i-1)d_0}^{\infty} \rho_{SNL}(z) w_{GL}(z) dz] + \int_{\delta_{SNL}}^{\infty} \rho_{SNL}(z) w_{SL}(z) dz \quad (3)$$

The former term refers to the vdW interaction between liquid and graphene sheets and the latter to the interaction between liquid and the solid substrate. However, the density of

liquid, $\rho_{SNL}(z)$ was defined where z=0 referred to the top of N-layer graphene, while $w_{GL}(z)$ was defined on the i-th graphene and $w_{SL}(z)$ was defined on the solid substrate. Therefore the coordinate systems needed to be modified for compatibility and we fixed the equation as follow.

$$\Phi_{SNL} = \sum_{i=1}^{N}[\int_{\delta_{GL}+(i-1)d_0}^{\infty} \rho_{SNL}(z-(i-1)d_0)w_{GL}(z)dz] + \int_{\delta_{SNL}}^{\infty} \rho_{SNL}(z-\delta_{SNL}+\delta_{GL})w_{SL}(z)dz. \quad (4)$$

On the other hand, the vdW interaction per unit area between the liquid and the bare solid was correctly calculated by $\Phi_{\mathrm{SL}} = \int_{\delta_{SL}}^{\infty} \rho_{SL}(z)w_{SL}(z)dz$, where the liquid density profile is defined by $\rho_{\mathrm{SL}} = \rho_{L0} \exp\left[-\frac{w_{SL}(z)}{k_B T}\right]$ and δ_{SL} is the distance between the liquid and the bare solid.

Supplementary Note 2: On the Values of δ_{GS} and δ_{SL}

We set the solid-graphene equilibrium distance δ_{GS} as 3Å, while Shih et al. [2] assumed it as 3~5 Å, while existing studies reported 2~3.5 Å to be a realistic value [3-5]. Also, considering the typical vdW radius of a common solid and water molecules[6], we assume 3~4 Å for the distance δ_{SL} between the bare solid substrate and water, whereas it was set at an excessively large value of 5 Å in Shih et al.[2]. We note that $\mathrm{A_{CL}}$ must be tuned to make the contact angle of graphite equal to the experimental value of 86° every time we make corrections to the theory. Shih et al. used $\mathrm{A_{CL}} = 8.914eVA^6$, the math corrected model needs $\mathrm{A_{CL}} = 8.7\mathrm{eVA}^6$, and the math and density corrected model requires $\mathrm{A_{CL}} = 20.5eVA^6$.

Supplementary Note 3: Theory of Shih et al.[2] can Predict Experimental Data for Flat Substrates with Modified Values of θ_{GS}.

As illustrated in the results section, after a few mathematical errors are corrected, theory of Shih et al. cannot explain experimental results, predicting $\theta_{GS} = 0°$ for any solid with $\theta_S < 105°$ (**Fig. S2** red dotted). Still, if decreased value of $\delta_{SL} = 2.9 \sim 3.7$Å is used (the original theory of Shih et al. used 5Å for the value), the corrected theory predicts curves that can fit to experimental data set (**Fig. S2** black solid and black dotted). Therefore, one can say main conclusion of the theory of Shih et al. remains. However, even with the modification of δ_{SL}, the unrealistically high density of the liquid near the surface persists because the intermolecular interaction is neglected. Thus, the wetting theory still needs to be improved as suggested by this work.

Supplementary Note 4: Derivation of GRSR2 Wetting Model for Graphene Covered Rough Substrates

Flat monolayer graphene has its contact angle as 96° which is the upper bound of the contact angle of a flat graphene covered surface[2]. However, CVD transferred graphene can have considerable roughness[7, 8] affecting the contact angle of the graphene. Therefore, we consider an additional wetting mode in which rough graphene is non-conformally adhered to the rough substrate, which we will be referred as GRSR2 model (**Fig. S3a**). For the simplicity, we consider a periodic array of vertical posts as the texture of the rough surfaces as shown in **Fig. S3b**.

We define $\mathrm{r_G}$ $(\mathrm{r_S})$ as the roughness of graphene (substrate), and f_G (f_S) as the fraction of top area of graphene (substrate). As in the other wetting models, we assume that the characteristic length scale of roughness is much greater than the length scale of vdW potential. For Penetrate mode and Cassie-Baxter (CB) mode, lower part of graphene do not contribute to the vdW potential on the liquid. Also, the entire substrate lying below the graphene do not interact with the liquid. Thus, the contact angle θ'_{GS} is determined sole by the roughness parameters of the graphene, $\mathrm{r_G}$ and f_G as presented in **Table S1**.

The contact angle formula for the Wenzel mode is rather complicated (see Table S1). Conventional Wenzel model assumes uniform vdW potential between the liquid and the substrate over the effective area of solid $\mathrm{rA_0}$ where r is the roughness and $\mathrm{A_0}$ is the apparent area. However, in the GRSR2 model, the assumption does not hold for some portion of the rough graphene due to the substrate located below it. As illustrated in **Fig. S3a**, the graphene can be divided into two parts; a part directly adheres to the substrate (f_S) and the other part floats on the substrate $(1 - f_s)$. For the latter, the liquid only interact with the rough graphene, giving rise to the $(1 - f_s) r_G \cos\theta_G$ term in the right hand side of the equation. However, for

the former, the portion of the liquid at the bottom interacts with both graphene and the substrate, while the other portion in the vicinity of the side wall and top surface interacts with only the graphene. In other words, the vdW potential between the liquid and the graphene covered substrate is not uniform along the interface. For the surface texture in **Fig S3b**, one can derive the relationship between r, f and α,h as $r = 1 + 4\alpha h$ and $f = \alpha^2$. As mentioned repeatedly, because of the length scale difference between the roughness and the vdW potential, the liquid near the most part of the side walls ($4\alpha h = r - 1$) and the upper part ($\alpha^2 = f$) interact only with the graphene, giving rise to the term $f_S(r_G + f_G - 1)cos\theta_G$. The bottom part ($1 - \alpha^2 = 1 - f$) interact with the both graphene and substrate, resulting in the $f_S(1 - f_G)cos\theta_{GS}$ contribution. The result can be generalized for the posts with arbitrary cross section having vertical side walls. By combining three contributions, we can derive the formula for Wenzel mode in **Table S1**. We note that the formula for Wenzel does not hold if the side wall of the post is inclined.

Due to the roughness of the graphene that increases effective area of graphene, the upper bound of GRSR2 model is higher than the upper bound of the GFSR model, i.e. the contact angle of flat monolayer graphene, as shown in **Fig. S3c**.

	GRSR2
Penetrate mode	$\cos\theta'_{GS} = f_G\cos\theta_G - f_G + 1$
Wenzel mode	$\cos\theta'_{GS} = f_S[(r_G + f_G - 1)cos\theta_G + (1 - f_G)cos\theta_{GS}] + (1 - f_S)r_G cos\theta_G$
Cassie Baxter mode	$\cos\theta'_{GS} = f_G\cos\theta_G + f_G - 1$

Table S1. | Contact angles of the GRSR2 model due to the wetting modes

References

1. Ingebrigtsen, T. & Toxvaerd, S. Contact angles of Lennard-Jones liquids and droplets on planar surfaces. *Journal of Physical Chemistry C* **111**, 8518-8523 (2007).
2. Shih, C.J. et al. Breakdown in the wetting transparency of graphene. *Phys Rev Lett* **109**, 176101 (2012).
3. Giovannetti, G. et al. Doping graphene with metal contacts. *Phys Rev Lett* **101**, 026803 (2008).
4. Giovannetti, G., Khomyakov, P.A., Brocks, G., Kelly, P.J. & van den Brink, J. Substrate-induced band gap in graphene on hexagonal boron nitride: *Ab initio* density functional calculations. *Physical Review B* **76**, 073103 (2007).
5. Xu, Z. & Buehler, M.J. Interface structure and mechanics between graphene and metal substrates: a first-principles study. *J Phys Condens Matter* **22**, 485301 (2010).
6. Bondi, A. van der Waals Volumes and Radii. *The Journal of Physical Chemistry* **68**, 441-451 (1964).
7. Reina, A. et al. Large area, few-layer graphene films on arbitrary substrates by chemical vapor deposition. *Nano Lett* **9**, 30-5 (2009).
8. Srivastava, A. et al. Novel Liquid Precursor-Based Facile Synthesis of Large-Area Continuous, Single, and Few-Layer Graphene Films. *Chemistry of Materials* **22**, 3457-3461 (2010).
